# Normal weight obesity, circulating biomarkers and risk of breast cancer: a prospective cohort study and meta-analysis

**DOI:** 10.1038/s41416-024-02906-1

**Published:** 2024-11-28

**Authors:** Wenjie Wang, Xiaoyan Wang, Ying Jiang, Yingying Guo, Peifen Fu, Wei He, Xiaohua Fu

**Affiliations:** 1 Department of Nutrition and Food Hygiene, Children’s Hospital, Zhejiang University School of Medicine, National Clinical Research Center for Child Health, Hangzhou, Zhejiang China; 2 Chronic Disease Research Institute, School of Public Health, School of Medicine, Zhejiang University, Hangzhou, Zhejiang China; 3 Department of Breast Surgery, The First Affiliated Hospital, School of Medicine, Zhejiang University, Hangzhou, Zhejiang China; 4 Binjiang Institute of Zhejiang University, Hangzhou, Zhejiang China; 5 Reproductive Medicine Center, Department of Reproductive Endocrinology, Zhejiang Provincial People’s Hospital, Affiliated People’s Hospital, Hangzhou Medical College, Hangzhou, Zhejiang China

**Keywords:** Breast cancer, Public health, Weight management

## Abstract

**Background:**

Individuals with normal weight obesity (NWO) often escape the attention of healthcare providers who may assume that a normal body mass index (BMI) correlates with low health risks. However, it remains unknown whether NWO increases the risk of breast cancer.

**Methods:**

This study included 22,257 and 52,506 pre- and postmenopausal females with normal BMI in the UK Biobank. NWO was defined as participants with a normal BMI (18.5–24.9 kg/m2) and an excess percent body fat (PBF > 33.3%). Cox proportional hazard models were used to investigate the associations of NWO and NWO-related biomarkers with incident breast cancer.

**Results:**

NWO was not associated with premenopausal breast cancer, whereas it was associated with a higher risk of postmenopausal breast cancer (hazard ratio = 1.19, 95% CI: 1.08–1.31). In our meta-analysis, per 5-unit increment in percent body fat level was linked to a 15% (95% CI: 10–19%) elevated risk of postmenopausal breast cancer in females with normal BMI. Stratified analyses showed a stronger positive association in females with higher genetic risk. In our NWO-biomarkers analyses, NWO was linked to 34 identified biomarkers, of which three inflammation markers (monocyte count, neutrophil count, and C-reactive protein), and one ketone body metabolite (β-Hydroxybutyrate) also indicated a positive association with postmenopausal breast cancer.

**Conclusions:**

NWO is associated with an increased risk of postmenopausal breast cancer, indicating that relying solely on BMI neglects the higher risk faced by non-obese postmenopausal women.

## Introduction

Obesity is recognized as a risk factor for at least 13 different types of cancer [1]. These epidemiological studies commonly use body mass index (BMI) to categorise body size into normal, overweight, or obese categories [2, 3]. However, BMI primarily measures overall obesity and does not accurately reflect body fat distribution [4, 5]. Consequently, individuals with a normal BMI might still be at increased disease risk due to excess body fat, a condition referred to as normal weight obesity (NWO) [6].

Previous two studies have identified a positive correlation between elevated body fat levels and an increased risk of postmenopausal breast cancer in women with normal BMI [7, 8]. However, the exploration of normal weight obesity (NWO) is crucial compared to continuous body fat levels, as it identifies a specific high-risk population for breast cancer, thereby enhancing risk stratification and personalized intervention strategies. Although obesity is a well-established risk factor for postmenopausal breast cancer [9, 10], it appears to have a protective effect against premenopausal breast cancer [11, 12]. Thus, it is crucial to determine if NWO similarly influences the risk of premenopausal breast cancer. Moreover, the potential role of lifestyle factors, female-specific variables, and genetic predispositions in modifying the relationship between NWO and breast cancer risk remains to be clarified.

The biological mechanisms linking NWO and breast cancer are not fully understood. Recent studies have shown that adipocyte hypertrophy in women with normal BMI may increase aromatase and leptin levels, potentially raising breast cancer risk [13]. However, other significant factors such as inflammation, oxidative stress, and metabolic disruptions—which are also key features for adiposity and breast cancer—have received less attention [14–17]. This leads to our hypothesis that these markers might play a role in the association between NWO and breast cancer.

Using data from the UK Biobank, this study aims to: (1) thoroughly examine the association of NWO with both pre- and postmenopausal breast cancer; (2) explore whether certain variables could modify the relationship between NWO and breast cancer, and (3) identify potential circulating biomarkers that could link NWO to breast cancer risk. To provide even more solid evidence, we also conducted a meta-analysis, including results from our study and two other cohort studies.

## Methods

### Study population and design

The UK Biobank study protocol is available to the public. This study is a large population-based cohort study that involves approximately 500,000 participants of diverse ethnicities aged between 40 to 69 years in 2006–2010 across the UK [18]. At baseline, participants completed self-reported touchscreen questionnaires, underwent physical examinations, and provided biological samples for various laboratory assays. The study obtained ethical approval from the Multicenter Research Ethics Committee, the National Information Governance Board for Health and Social Care in England and Wales, and the Community Health Index Advisory Group in Scotland. All participants provided their informed written consent before participation. This study was conducted using the UK Biobank resource under application number 69972.

In the present study, we excluded 229,084 male participants; 167,688 underweight females (BMI < 18.5 kg/m^2^), overweight or obese (BMI ≥ 25 kg/m^2^); 13,325 had a history of cancer at baseline; 1180 without percent body fat (PBF) data; 11,477 had missing data on menopausal information; leaving 79,658 females with normal BMI (18.5 < BMI ≤ 24.9 kg/m^2^). Among baseline self-reported premenopausal females, we set the cut-off point for the end of follow-up at 50 years of age. Thus, we defined premenopausal cases as those arising in women younger than 50 years [19]. Finally, 22,257 premenopausal and 52,506 postmenopausal females with normal BMI were included in the current analysis, respectively (Supplementary Fig. 1).

### Definition of Normal weight obesity

Normal weight obesity was defined as participants with a normal BMI (18.5–24.9 kg/m^2^) but an excess percent body fat (>33.3%) [20, 21]. Body mass index (BMI) was calculated as weight divided by height squared (kg/m^2^). PBF was measured using the Tanita BC418MA body composition analyser (total fat mass divided by the total body mass). Normal weight lean (NWL) was defined as participants with a normal BMI and a normal PBF category (≤33.3%).

### Ascertainment of breast cancer incidence

The outcome of this study was breast cancer incidence. In the UK Biobank, cancer diagnoses were ascertained through linkage to national cancer registries. The follow-up time refers to the period from baseline enrollment to the first diagnosis of breast cancer, death, or end of follow-up (30 June 2021 for Scotland and 31 December 2020 for England and Wales). The 9th and 10th Revisions of the International Classification of Diseases were used to code breast cancer (ICD-9:174, ICD-10:C50).

### Measurement of oxidative stress, inflammation, and metabolome

30 blood biochemistry markers and 31 blood cell counts were measured at the UK Biobank initial assessment, they performed detailed quality control (QC) and correction for technical outliers. Details for blood analytes and counts have been described previously [22, 23]. The inflammation marker used in this study included peripheral blood cell counts for neutrophils, lymphocytes, platelets, and C-reactive protein (CRP), while the oxidative stress markers included total bilirubin and gamma-glutamyltransferase.

A high-throughput nuclear magnetic resonance (NMR) metabolomics spectroscopy was used to undertake metabolomics profiling on baseline plasma samples from approximately 120,000 randomly selected UK Biobank participants [24]. In brief, 168 directly measured metabolic biomarkers, including lipoproteins, lipids, fatty acids, amino acids, and other low-molecular-weight metabolites such as ketone bodies and glycolysis metabolites were simultaneously quantified. For the lipoprotein subclass, we mainly focus on lipoprotein particle concentration in 14 subclasses. Finally, a subset of 85 metabolites were included in the current analysis (Supplementary Table 1).

### Assessment of covariates

Demographic information and sex-specific factors were collected using a touch-screen questionnaire at baseline assessment. Demographic information included age at recruitment (years), race (whites and others), smoking status (never, ever, and current), drinking status (never, ever, and current), physical activity (low, medium and high), education attainment (O levels/GCSEs or equivalent, A/AS levels or equivalent, university and others) and socioeconomic status (based on tertiles of Townsend deprivation index); female-specific factors including family history of breast cancer, history of mammograms screening, age at menarche, age at first live birth, number of births, hormone replacement therapy (HRT) (only postmenopausal), ever use of oral contraceptives and age at menopause (only postmenopausal). Standard polygenic risk score (PRS) for breast cancer was available in the UK Biobank (data field 26220 - standard PRS for breast cancer) [25]. Then, the PRS was categorised into low (lowest tertile), intermediate (second tertile), and high (highest tertile) genetic risk.

### Statistical analysis

Either Student’s *t*-test or Chi-square test was used to compare the differences in baseline characteristics in the NWL and NWO groups by menopausal status. The Cox proportional hazards model was employed to evaluate the hazard ratios (HRs) and corresponding 95% confidence intervals (CIs) for the association between NWO and breast cancer incidence. The proportional hazards assumption was examined based on the Schoenfeld residuals. Analyses were performed separately by menopausal status. Missing values were grouped as a separate category (unknown) for each covariate.

We conducted a meta-analysis based on available cohort studies and this study to examine the association of PBF with postmenopausal breast cancer in females with normal BMI. To quantify this association, the HRs (95%CIs) per 5-unit increment in PBF level were calculated for each cohort according to the methods developed by Greenland and colleagues [26]. We used I^2^ statistics to assess heterogeneity across studies, and the results were pooled using random-effects models when I^2^ > 50%; otherwise, a fixed-effects model was used. Details were provided in the supplementary methods.

We conducted stratified analyses to examine whether the association of NWO with postmenopausal breast cancer was modified by genetic risk and other variables. The product terms were included to test the significance of the multiplicative interaction. The additive interaction was evaluated using the relative excess risk due to the interaction (RERI) and the attributable proportion due to the interaction (AP).

All biomarkers were nature log-transformed and standardized by Z-score. Orthogonal partial least squares discrimination analysis (OPLS-DA) was built to evaluate the overall distribution and distinguish the differential metabolites between the NWL and NWO groups using the R *ropls* package. The cross-validated analysis of variation (CV-ANOVA) was utilized to assess the model validity. We employed the multivariable linear regression and Cox regression models to evaluate the associations among NWO, circulating biomarkers (metabolism, oxidative stress, and inflammation), and incident breast cancer. In addition, we included circulating biomarkers as covariates in the fully adjusted model to explore their potential roles in the association between NWO and breast cancer.

Finally, we performed two sensitivity analyses to examine the robustness of the observed results. First, we excluded the women whose breast cancer was diagnosed within two years of follow-up. Second, we restricted the analysis to participants with complete covariate data for comparison with the main results. Third, we applied new definitions for NWO using PBF thresholds of ≥30%, 35%, and 37% and evaluated their association with breast cancer. All statistical analyses were performed using R software version 4.2.2. Unless otherwise stated, two-sided *P* < 0.05 were considered statistically significant.

## Results

### Percent body fat and breast cancer incidence by BMI category

**Fig. 1:**
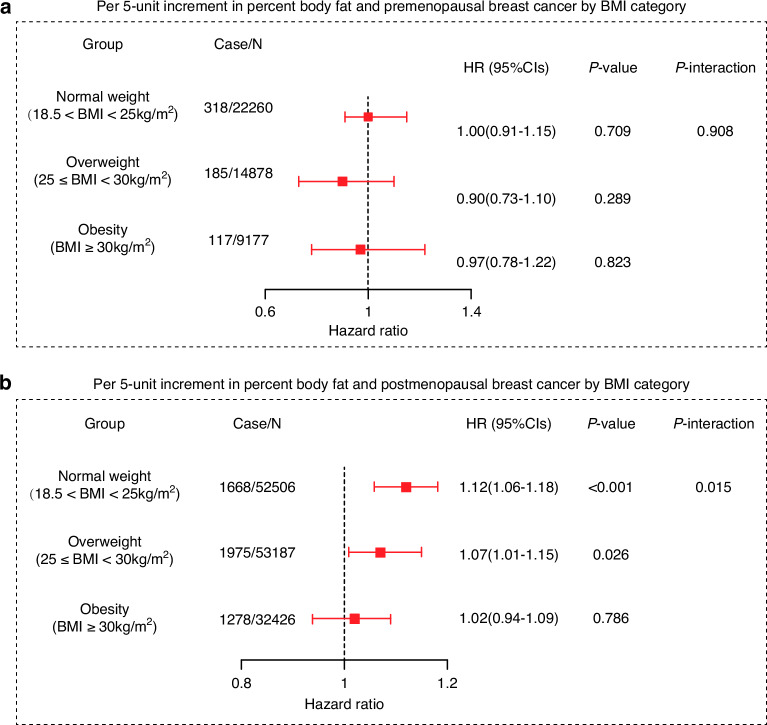
Association of per 5-unit increment in percent body fat with breast cancer incidence by BMI category. **a** Percent body fat and premenopausal breast cancer by BMI category. **b** Percent body fat and postmenopausal by BMI category. The Wald test was employed in the Cox proportional hazards model to calculate the *P* value for hazard ratios across different BMI categories. The *P* value for the product terms between percent body fat and BMI category was estimated by testing the significance of multiplicative interaction terms in the models.

Figure 1 shows the relationship between each 5-unit increase in percent body fat and pre- and postmenopausal breast cancer across different body mass index categories. There was no significant statistical link between percent body fat and premenopausal breast cancer. In contrast, a pronounced positive association was observed between the percent body fat and the risk of postmenopausal breast cancer, particularly those with a normal body mass index.

### Normal weight obesity and breast cancer incidence, association analysis

**Fig. 2:**
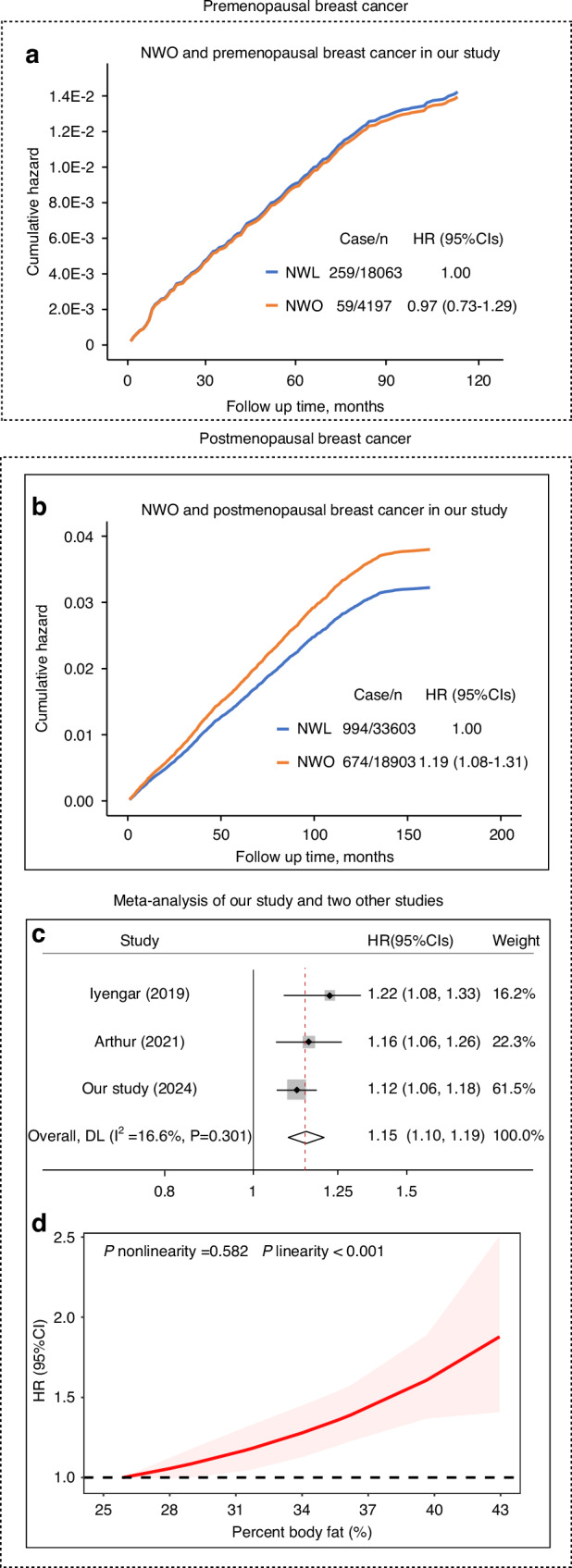
Normal weight obesity (NWO) and premenopausal and postmenopausal breast cancer. **a** Normal weight obesity (NWO) and premenopausal breast cancer. **b** NWO, and postmenopausal breast cancer. Cox proportional hazard models were employed to calculate the hazard ratios (HRs) and 95% confidence intervals (CIs). **c** Meta-analysis for the associations between per 5-unit increment in percent body fat (PBF) and postmenopausal breast cancer among females with normal BMI. **d** Dose-response meta-analysis of PBF level with postmenopausal breast cancer among females with normal BMI. The *P*-value was estimated using maximum likelihood estimation with the R package *dosresmeta*.

Supplementary Table 2 shows the baseline characteristics of non-obese participants by menopausal status. Consistently, we did not observe a statistically significant association between NWO and premenopausal breast cancer (Fig. 2a). However, NWO was significantly associated with an elevated risk of postmenopausal breast cancer. Compared with the normal weight lean group, the multivariate-adjusted hazard ratio (95%CIs) in the NWO group was 1.19 (1.08–1.31) (Fig. 2b).

Supplementary Table 3 shows the population-attributable risk proportions (PARP) for postmenopausal breast cancer. The PARP for normal weight obesity was 6.40 (95% CI: 2.80–10.04), exceeding the PARPs for family history of breast cancer (4.81; 95% CI: 2.98–6.88), nulliparity (2.15; 95% CI: −1.67–6.67), and oral contraceptive use (3.83; 95% CI: −6.81–13.10). Moreover, this estimate is also comparable to that for hormone replacement therapy (HRT), which had a PARP of 6.83 (95% CI: 2.23–11.34).

### Normal weight obesity and postmenopausal breast cancer, meta-analysis

Our meta-analysis incorporated two prior studies along with our data to pool the hazard ratio for postmenopausal breast cancer. We employed a fixed-effects model, justified by low heterogeneity (I^2^ = 16.6%). Among women with a normal body mass index, the pooled hazard ratio for postmenopausal breast cancer was 1.15 (95% CI: 1.10–1.19) for each 5-unit increase in percent body fat (Fig. 2c). Furthermore, dose-response meta-analysis confirmed a significant linear relationship between percent body fat and postmenopausal breast cancer risk in women with normal body mass index (Fig. 2d).

### Normal weight obesity and postmenopausal breast cancer, stratified analysis

Subgroup analyses demonstrated that the association between NWO and postmenopausal breast cancer was not modified by stratified variables except for the polygenic risk score (*P*-interaction = 0.027). The association was particularly strong among females with a high genetic risk (Table 1). Furthermore, a significant additive interaction was noted between NWO and genetic risk, leading to an increased overall risk of breast cancer as both NWO exposure and polygenic risk score levels rose. The relative excess risk due to the interaction (RERI) and the attributable proportion (AP) were 0.79 (95% CI: 0.25–1.33) and 0.19 (95% CI: 0.07–0.32) (Supplementary Fig. 2).

**Table 1:** Normal weight obesity (NWO) and incident postmenopausal breast cancer by subgroups in the UK Biobank.

Subgroups	No. of participants	NWL	NWO	*P*-interaction^a^
Age				0.702
<60 years	24,072	1.00	1.15 (0.98–1.34)	
≥60 years	28,434	1.00	1.22 (1.07–1.38)	
Ethnic				0.715
White	50,526	1.00	1.19 (1.08–1.32)	
Others	1980	1.00	1.04 (0.59–1.80)	
Smoking status				0.309
Never	31,631	1.00	1.21 (1.06–1.38)	
Ever	16,299	1.00	1.19 (1.00–1.40)	
Current	4428	1.00	1.08 (0.77–1.51)	
Drinking status				0.304
Never	2591	1.00	1.46 (0.91–2.34)	
Ever	1626	1.00	1.04 (0.60–1.82)	
Current	48,243	1.00	1.17 (1.06–1.30)	
Physical activity				0.396
Low	5621	1.00	1.33 (1.01–1.74)	
Medium	17,713	1.00	1.08 (0.92–1.28)	
High	18,052	1.00	1.21 (1.01–1.45)	
Educational level				0.927
College or University degree	18,270	1.00	1.14 (0.96–1.36)	
A/AS levels or equivalent	6122	1.00	1.24 (0.95–1.64)	
O levels/GCSEs or equivalent	11,741	1.00	1.14 (0.94–1.40)	
Others	11,5577	1.00	1.28 (1.00–1.45)	
Townsend Deprivation Index				0.780
Low	17,483	1.00	1.20 (1.03–1.40)	
Intermediate	17,479	1.00	1.33 (1.13–1.58)	
High	17,486	1.00	1.00 (0.82–1.22)	
Polygenic risk score				**0.027**
Low	17,019	1.00	1.11 (0.87–1.42)	
Intermediate	17,019	1.00	1.06 (0.87–1.28)	
High	17,024	1.00	1.32 (1.13–1.48)	
History of mammograms				0.855
Yes	49,797	1.00	1.17 (1.06–1.29)	
No	2679	1.00	1.64 (0.93–2.87)	
HRT use				0.358
Yes	23,980	1.00	1.21 (1.06–1.40)	
No	28,409	1.00	1.15 (1.00–1.32)	
Number of births				0.743
Nulliparous	9594	1.00	1.21 (0.96–1.52)	
<3	30,934	1.00	1.14 (1.00–1.29)	
≥3	11,978	1.00	1.33 (1.07–1.66)	
Age at menarche, years				0.448
<12	9370	1.00	1.21 (0.96–1.53)	
12–13	22,437	1.00	1.24 (1.07–1.45)	
≥14	20,669	1.00	1.12 (0.96–1.31)	
Age at menopause, years				
<50	20,840	1.00	1.30 (1.10–1.53)	0.772
50–55	24,969	1.00	1.09 (0.95–1.26)	
≥55	6687	1.00	1.26 (0.99–1.61)	

Cases with missing information were excluded.

*NWL* normal weight lean, *HRT* Hormone replacement therapy.

^a^Models were adjusted for age, gender, race, smoking status, drinking status, physical activity, education attainment, socioeconomic status, family history of breast cancer, history of mammograms, age at menarche, age at first live birth, number of births, hormone replacement therapy (HRT) use, oral contraceptives and age at menopause, with the exception of stratifying factors.

### Normal weight obesity, inflammation and oxidative stress markers, and post-menopausal breast cancer

**Fig. 3:**
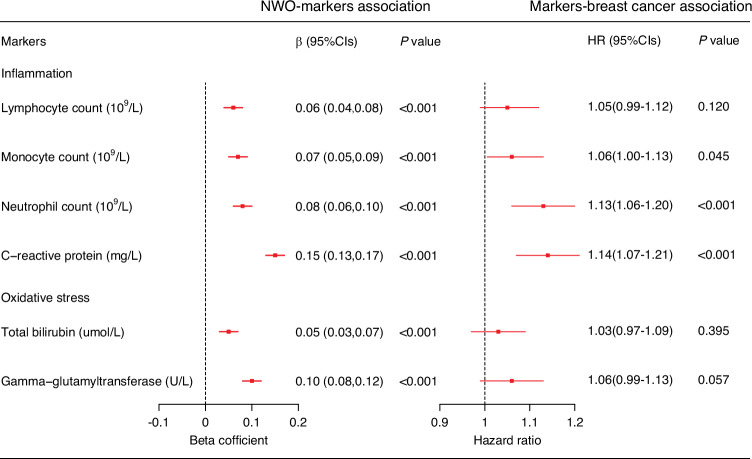
Normal weight obesity (NWO), inflammatory and oxidative stress markers, and incident postmenopausal breast cancer. The *T*-test in the linear regression model and the Wald test in the Cox proportional hazards model were employed to calculate the *P* values of the beta coefficient and hazard ratio, respectively. Inflammatory and oxidative stress markers were naturally log-transformed and standardized by z-score.

After adjusting for multiple variables, NWO was significantly associated with elevated levels of all measured inflammation and oxidative stress markers (*P* < 0.001). Specifically, inflammation markers such as monocyte count, neutrophil count, and C-reactive protein were linked to an increased risk of postmenopausal breast cancer (Fig. 3). However, no statistically significant relationship was observed between oxidative stress markers and postmenopausal breast cancer, although a trend toward a positive association was noted. When we added monocyte count, neutrophil count, and C-reactive protein to the fully adjusted models, the effect sizes of NWO on postmenopausal breast cancer were attenuated by 3.74%, 11.23%, and 19.25%, respectively (Supplementary Table 4).

### Normal weight obesity, plasma metabolites, and postmenopausal breast cancer

**Fig. 4:**
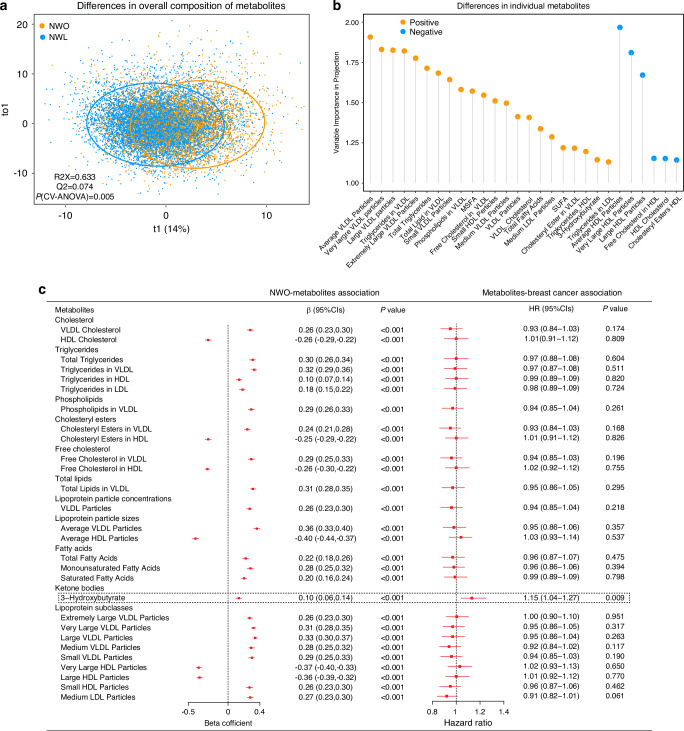
Normal weight obesity (NWO), plasma metabolites, and incident postmenopausal breast cancer. **a** The differences in the overall composition of serum metabolites between the normal weight lean (NWL) and NWO groups. The cross-validated analysis of variation was utilized to assess the *P* value of model validity. **b** Differential metabolites identified through variable importance of the projection (VIP) values (VIP > 1). **c** β-Hydroxybutyrate was associated with both NWO and breast cancer. The *T*-test in the linear regression model and the Wald test in the Cox proportional hazards model were employed to calculate the *P* values of the beta coefficient and hazard ratio, respectively. Metabolite concentrations were naturally log-transformed and standardized by *z*-score.

In the metabolomics analysis, we found significant differences in the overall composition of serum metabolites between the normal-weight lean and NWO groups among postmenopausal females (*P*-CV-ANOVA < 0.001) (Fig. 4A). We identified 28 differential metabolites between the two groups that might serve as NWO-related candidate metabolites, including 12 lipid species, 12 lipoproteins, 3 fatty acids, and 1 ketone body (Fig. 4B).

After multivariable adjustment, NWO was significantly associated with all above identified differential metabolites (all *P* < 0.001). We did not observe the statistically significant association between these metabolites and postmenopausal breast cancer, except for β-Hydroxybutyrate. β-Hydroxybutyrate was associated with an increased risk of postmenopausal breast cancer, the multivariable-adjusted hazard ratio was 1.15 (95% CI: 1.04–1.27) (Fig. 4c). Moreover, the effect size of NWO on postmenopausal breast cancer was attenuated by 9.09%, when adding β-Hydroxybutyrate to the fully adjusted model (Supplementary Table 4).

### Sensitivity analyses

In postmenopausal females, excluding participants diagnosed with breast cancer within 2 years of follow-up (Supplementary Table 5), excluding participants who had incomplete covariate data (Supplementary Table 6) and redefining NWO as percent body fat ≥30%, ≥35%, and ≥37% provided similar results as in the main analysis (Supplementary Table 7).

## Discussion

In this prospective cohort study, NWO was not related to premenopausal breast cancer but was related to an increased risk of postmenopausal breast cancer. A higher genetic risk may exacerbate the detrimental effect of NWO on postmenopausal breast cancer. In addition, inflammatory pathways and ketone body metabolism may play a role in the association between NWO and postmenopausal breast cancer.

BMI is currently the primary tool for health risk classification, but its sensitivity in assessing fat levels is limited [27]. In this study, we calculated the NWO prevalence to be 31.4% among participants with normal BMI. Similarly, prior literature reported that the prevalence of NWO was 31.3% and 27.8% among females aged ≥ 60 years in the US and females aged 45-75 years in Swedish, respectively [28, 29]. These data emphasize the widespread prevalence of NWO in older women, suggesting that NWO is an important but neglected sub-population that needs more investigation.

Our findings indicated that NWO is linked to a 19% increased risk of post-menopausal breast cancer. To validate these results, we expanded our analysis by conducting a meta-analysis that integrated our data with two other cohort studies. This approach significantly enhanced the sample size by 10 to 25 times compared to previous studies, providing a more robust and precise estimate [7, 8]. The consolidated data showed that for women with a normal BMI, every 5-unit rise in percent body fat corresponded to a 15% increase in the risk of developing postmenopausal breast cancer. This level of risk is on par with recognized risk factors such as hormone replacement therapy (HRT) and nulliparity, which are associated with a 15% (95% CI, 1.09–1.21) and 13% (95% CI, 1.00–1.28) increased risk, respectively [30, 31]. These findings emphasize the importance of early management of body fat levels to reduce the risk of postmenopausal breast cancer in women who are otherwise considered to be of normal weight.

We calculated the population attributable risk proportion (PARP) of NWO for postmenopausal breast cancer as 6.40 (95% CI, 2.80–10.04). This estimate notably exceeds the PARP for a family history of breast cancer, which was 4.81 (95% CI, 2.98–6.88) in our study and 5.10 (95% CI, 2.10–7.40) in a previous breast cancer surveillance study [32]. Annually, about 1.4 million postmenopausal breast cancer cases are diagnosed worldwide [33], highlighting the substantial impact of NWO, which may contribute to tens of thousands of these cases each year. Therefore, it is crucial to inform postmenopausal women that a normal BMI does not reliably indicate low breast cancer risk.

Beyond environmental factors, genetics are also a strong explanatory factor for breast cancer [34]. One Nordic cohort study suggested that heritable factors are responsible for 27% of the total risk of breast cancer [35]. Gene-environment interaction studies may offer novel perspectives for precise cancer interventions [36]. In this study, our results showed that the observed positive link was stronger in women with high genetic risk. In addition, we observed that 19% of breast cancer risk could be attributed to additive interactions, suggesting the combined effect of NWO exposure and high genetic risk was greater than the sum of the two individual effects. Given these results, it can be implied that postmenopausal women who are genetically predisposed to breast cancer would benefit more from lowering body fat, which emphasizes personalized preventive strategies in breast cancer management.

Prior studies have consistently shown that obesity is a risk factor for postmenopausal breast cancer. This higher risk may stem from hormonal mechanisms [37]. Oestrogen is mainly synthesized in adipose tissue among postmenopausal women. The higher fat levels were associated with higher levels of serum estrone and estradiol and lower levels of sex hormone-binding globulin, which is linked to an increased risk of breast cancer [38, 39]. In addition to that, our study provided another two potential mechanisms.

In our study, the association of NWO with postmenopausal breast was attenuated with further adjustment of inflammation markers (monocyte count, neutrophil count, and CPR) and ketone body (β-Hydroxybutyrate), suggesting that the detrimental effect of NWO might partially through the inflammatory pathway and ketone body metabolism. Firstly, previous research indicates that individuals with NWO exhibit higher levels of inflammation compared to those with NWL [40]. Secondly, inflammatory factors and their receptors are known to play crucial roles in various stages of breast cancer progression, including cell proliferation, differentiation, tumour metastasis, and angiogenesis [41]. Additionally, many soluble cytokines released by inflammatory leucocytes are implicated in breast cancer [42].

To our knowledge, this is the first study to report that β-Hydroxybutyrate may play a role in the positive association between NWO and postmenopausal breast cancer. The detailed mechanism remains to be elucidated but may involve ketone body metabolism and the expression of oncogenes. β-Hydroxybutyrate serves as a critical ketone body in the human body [43]. Hepatocytes synthesize ketone bodies via acetyl coenzyme A derived from fatty acid β-oxidation, where adipose tissue-derived fatty acids act as crucial precursors [44]. Thus, the elevated fat levels in the NWO group may account for increased circulating levels of β-Hydroxybutyrate. Moreover, Huang et al. reported that treatment with β-hydroxybutyrate enhanced the tumorigenic properties of breast cancer cells in the mouse xenograft model [45]. Microarray analysis further revealed that β-Hydroxybutyrate might upregulate tumour-promoting genes through chromatin epigenetic modifications, thereby promoting breast tumorigenicity [45]. Taken together, both inflammatory pathways and ketone body metabolism are involved in the development of postmenopausal breast cancer in women with NWO.

Previous reports suggested obesity might protect against premenopausal breast cancer, but our findings show no association between NWO and premenopausal breast cancer. Two potential explanations for this discrepancy are age and body fat variation. A previous multicenter study involving 758,592 women suggested a stronger protective association between obesity and premenopausal breast cancer in women aged 18 to 24 than those between 45 to 54 years [11]. Similarly, BMI at 18 years was found to be the strongest predictor for premenopausal breast cancer in the Nurses’ Health Study II [46]. These results underscore the critical role of early adult body size in premenopausal breast cancer development. In contrast, the UK Biobank recruits women predominantly over 40, many nearing menopause. Thus, the shorter follow-up period and fewer cases might account for the nonsignificant results. In addition, the mean PBF was 35.36% for NWO, 38.36% for overweight, and 44.80% for obese categories in our study. The significant protective effects might not be observable at relatively lower body fat levels. Future research, comprising premenopausal women from a diverse age range, is warranted to confirm our results.

The findings of this study have significant clinical implications for the management and prevention of postmenopausal breast cancer. Our results indicated that normal weight obesity (NWO) is associated with an elevated risk of postmenopausal breast cancer, highlighting the necessity for healthcare providers to look beyond traditional BMI classifications when assessing cancer risk. This underscored the importance of incorporating comprehensive body composition assessments into routine clinical evaluations to better identify at-risk individuals, particularly among those with normal BMI. Moreover, the observed association of NWO with inflammatory and oxidative stress markers suggested that targeted interventions aimed at reducing body fat and improving metabolic health could be beneficial. Specifically, lifestyle modifications, such as increased physical activity and dietary adjustments, should be emphasized for women identified as having NWO, especially those with a high genetic predisposition. By adopting a more nuanced approach to cancer risk assessment and management, clinicians can enhance prevention strategies and potentially reduce the incidence of postmenopausal breast cancer among this vulnerable population.

### Strengths and limitations

Strengths of this study included the larger sample size, long-term follow-up, consideration of female-specific factors, and integration of genetic and metabolomics data. Nevertheless, several limitations need acknowledgement. First, we cannot exclude the possibility of misclassification of breast cancer diagnosis due to the nature of registry data. Second, we cannot evaluate the association of NWO with breast cancer by tumour stage and hormone-receptor subtype, since they were not available in the UK Biobank. Third, we failed to obtain accurate information on premenopausal breast cancer, as we used age as a proxy. Fourth, our results may not be representative of other races, given that more than 90% of females are predominantly of European descent.

## Conclusions

In summary, our study shows that NWO was linked to a higher risk of postmenopausal breast cancer, especially in women with high genetic risk. β-Hydroxybutyrate and inflammation markers emerge as potential contributors to this positive link. NWO is frequently neglected when merely using BMI as a predictor for postmenopausal breast cancer risk. Thus, postmenopausal women should be reminded that a normal BMI but higher body fat levels are actually associated with an increased risk of breast cancer.

## Supplementary information

Supplementary materials

## Data Availability

The data supporting the findings of the study are available on application to the UK Biobank (www.ukbiobank.ac.uk/).
