# Supplementary material for: Normal weight obesity, circulating biomarkers and risk of breast cancer: a prospective cohort study and meta-analysis
Source: Br J Cancer. 2024 Nov 28;132(2):203–11. doi: 10.1038/s41416-024-02906-1 (PMC11747320; doi:10.1038/s41416-024-02906-1)
Supplement: Supplementary file 1 — Supplementary materials [file 41416_2024_2906_MOESM1_ESM.docx]

**Supplementary methods**

**Inflammation and oxidative stress measurements**

30 blood biochemistry markers and 31 blood cell counts were measured at the initial assessment. The UK Biobank performed detailed quality control (QC) and correction for technical outliers. Details for blood analytes and counts are available at https:// biobank.ctsu.ox.ac.uk/crystal/crystal/docs/serum_biochemistry.pdf and https://biobank. ctsu.ox.ac.uk/crystal/crystal/docs/haematology.pdf, respectively. The inflammation marker used in this study included peripheral blood cell counts for neutrophils, lymphocytes, and platelets, while the oxidative stress markers included total bilirubin and gamma-glutamyltransferase.

**Meta-analysis**

We performed a systematic literature search of the PubMed, Web of Science, Embase, and Scopus databases for published cohort studies until 31 May 2023. The search strategies were as follows: (breast cancer incidence) AND (normal weight obesity OR body fat percentage (BFP) and normal weight obesity) AND (postmenopausal OR postmenopausal females). We included articles if they met the following criteria: 1) cohort design; 2) targeted females with normal weight; 3) baseline BFP levels were measured; 4) results were breast cancer incidence; 5) HRs and 95% CIs were provided; and 6) the confounders were adjusted between BFP level and breast cancer incidence. General characteristics extracted from each cohort study were as follows: first author’s name, publication year, sample size, follow-up year, outcomes, number of cases, HRs and 95% CIs, and confounders.

To quantify the dose-response association between BFP level and breast cancer incidence among normal-weight postmenopausal females, the unit of HRs (95%CIs) was standardized to per 5% increment in BFP level for each cohort study according to the methods developed by Greenland and colleagues. For studies reporting HRs by quantiles of BFP level, the midpoint of the lower and upper bounds was used as a proxy of the median. The widths of the open-ended categories were assumed at the same width as the adjacent interval. We used I^2^ statistics to assess heterogeneity across studies; study-specific results we pooled using random-effects models when I^2^ > 50%, otherwise, a fixed-effects model was used.

**Calculation of** **population attributable risk proportion (PARP)**

The PARP for normal weight obesity (NWO) was estimated using the following formula:

$${PARP}_{NWO}=\frac{P_{NWO}({RR}_{NWO}-1)}{(P_{NWO}\left[ {RR}_{NWO}-1 \right]+1)}$$

where P_NWO_ is the population prevalence of NWO, and RR_NWO_ is the relative risk of NWO. Following previous studies, we used confidence intervals from the RR for NWO to calculate upper and lower bounds for estimates of PARP.

**Supplementary Table 1.** Nuclear magnetic resonance (NMR) metabolites applied in the current study.

| field_id | Metabolite’s description used in the UK Biobank |
| --- | --- |
|  | **Cholesterol** |
| 23400 | Total Cholesterol |
| 23401 | Total Cholesterol Minus HDL-C |
| 23402 | Remnant Cholesterol (Non-HDL, Non-LDL -Cholesterol) |
| 23403 | VLDL Cholesterol |
| 23404 | Clinical LDL Cholesterol |
| 23405 | LDL Cholesterol |
| 23406 | HDL Cholesterol |
|  | **Triglycerides** |
| 23407 | Total Triglycerides |
| 23408 | Triglycerides in VLDL |
| 23409 | Triglycerides in LDL |
| 23410 | Triglycerides in HDL |
|  | **Phospholipids** |
| 23411 | Total Phospholipids in Lipoprotein Particles |
| 23412 | Phospholipids in VLDL |
| 23413 | Phospholipids in LDL |
| 23414 | Phospholipids in HDL |
|  | **Cholesteryl esters** |
| 23415 | Total Esterified Cholesterol |
| 23416 | Cholesteryl Esters in VLDL |
| 23417 | Cholesteryl Esters in LDL |
| 23418 | Cholesteryl Esters in HDL |
|  | **Free cholesterol** |
| 23419 | Total Free Cholesterol |
| 23420 | Free Cholesterol in VLDL |
| 23421 | Free Cholesterol in LDL |
| 23422 | Free Cholesterol in HDL |
|  | **Total lipids** |
| 23423 | Total Lipids in Lipoprotein Particles |
| 23424 | Total Lipids in VLDL |
| 23425 | Total Lipids in LDL |
| 23426 | Total Lipids in HDL |
|  | **Lipoprotein particle concentrations** |
| 23427 | Total Concentration of Lipoprotein Particles |
| 23428 | Concentration of VLDL Particles |
| 23429 | Concentration of LDL Particles |
| 23430 | Concentration of HDL Particles |
| 23431 | Average Diameter for VLDL Particles |
| 23432 | Average Diameter for LDL Particles |
| 23433 | Average Diameter for HDL Particles |
|  | **Other lipids** |
| 23434 | Phosphoglycerides |
| 23436 | Total Cholines |
| 23437 | Phosphatidylcholines |
| 23438 | Sphingomyelins |
|  | **Apolipoproteins** |
| 23439 | Apolipoprotein B |
| 23440 | Apolipoprotein A1 |
|  | **Fatty acids** |
| 23442 | Total Fatty Acids |
| 23443 | Degree of Unsaturation |
| 23444 | Omega-3 Fatty Acids |
| 23445 | Omega-6 Fatty Acids |
| 23446 | Polyunsaturated Fatty Acids |
| 23447 | Monounsaturated Fatty Acids |
| 23448 | Saturated Fatty Acids |
| 23449 | Linoleic Acid |
| 23450 | Docosahexaenoic Acid |
|  | **Amino acids** |
| 23460 | Alanine |
| 23461 | Glutamine |
| 23462 | Glycine |
| 23463 | Histidine |
| 23464 | Total Concentration of Branched-Chain Amino Acids |
| 23465 | Isoleucine |
| 23466 | Leucine |
| 23467 | Valine |
| 23468 | Phenylalanine |
| 23469 | Tyrosine |
|  | **Glycolysis related metabolites** |
| 23470 | Glucose |
| 23471 | Lactate |
| 23472 | Pyruvate |
| 23473 | Citrate |
|  | **Ketone bodies** |
| 23474 | 3-Hydroxybutyrate |
| 23475 | Acetate |
| 23476 | Acetoacetate |
| 23477 | Acetone |
|  | Fluid balance |
| 23478 | Creatinine |
| 23479 | Albumin |
|  | **Inflammation** |
| 23480 | Glycoprotein Acetyls |
|  | **Lipoprotein subclasses** |
| 23481 | Concentration of Chylomicrons and Extremely Large VLDL Particles |
| 23488 | Concentration of Very Large VLDL Particles |
| 23495 | Concentration of Large VLDL Particles |
| 23502 | Concentration of Medium VLDL Particles |
| 23509 | Concentration of Small VLDL Particles |
| 23516 | Concentration of Very Small VLDL Particles |
| 23530 | Concentration of Large LDL Particles |
| 23537 | Concentration of Medium LDL Particles |
| 23544 | Concentration of Small LDL Particles |
| 23551 | Concentration of Very Large HDL Particles |
| 23565 | Concentration of Medium HDL Particles |
| 23572 | Concentration of Small HDL Particles |

Abbreviation: HDL, high-density lipoprotein; LDL, low-density lipoprotein; VLDL, very low-density lipoprotein; IDL, intermediate-density lipoprotein

**Supplementary Table 2** Baseline characteristics of the normal weight lean (NWL) and normal weight obesity (NWO) groups by menopausal status in the UK biobank

|  | **Premenopausal** | |  | **Postmenopausal** | |
| --- | --- | --- | --- | --- | --- |
| **Characteristics ^b^** | **NWL**  **(N=17904)** | **NWO ^a^**  **(N=4353)** |  | **NWL ^a^**  **(N=33603)** | **NWO ^a^**  **(N=18903)** |
| Age, years | 44.53(2.66) | 44.66(2.69) |  | 59.34(5.49) | 60.34(5.37) |
| Ethnic, % |  |  |  |  |  |
| White | 16647(93.0) | 3971(91.2) |  | 32372(96.3) | 18154(96.0) |
| Others | 1257(7.0) | 382(8.9) |  | 1231(3.7) | 749(4.0) |
| Smoking, % |  |  |  |  |  |
| Never | 11705(65.5) | 2881(66.4) |  | 20394(60.8) | 11237(59.7) |
| Ever | 4287(24.0) | 1057(24.4) |  | 10151(30.3) | 6148(32.6) |
| Current | 1887(10.6) | 402(9.3) |  | 2986(8.9) | 1442(7.7) |
| Missing | 25 | 13 |  | 72 | 76 |
| Drinking, % |  |  |  |  |  |
| Never | 659(3.7) | 214(4.9) |  | 1627(4.8) | 964(5.1) |
| Ever | 429(2.4) | 84(1.9) |  | 1091(3.3) | 535(2.8) |
| Current | 16806(93.9) | 4052(93.1) |  | 30851(91.9) | 17392(92.1) |
| Missing | 10 | 3 |  | 34 | 12 |
| Physical activity |  |  |  |  |  |
| Low | 2034(13.4) | 719(19.7) |  | 3233(12.1) | 2388(16.3) |
| Medium | 6328(41.7) | 1665(45.7) |  | 11158(41.8) | 6555(44.6) |
| High | 6826(44.9) | 1260(34.6) |  | 12310(46.1) | 5742(39.1) |
| Missing | 2716 | 709 |  | 6902 | 4218 |
| Educational level, % |  |  |  |  |  |
| College or University degree | 8472(49.5) | 1902(44.2) |  | 12539(37.9) | 5731(30.8) |
| A levels/AS levels or equivalent | 2697(15.3) | 653(15.2) |  | 4041(12.2) | 2081(11.2) |
| O levels/GCSEs or equivalent | 3683(20.8) | 1062(24.7) |  | 7205(21.8) | 4536(24.4) |
| Others | 2551(14.4) | 683(15.9) |  | 9315(28.1) | 6262(33.6) |
| Missing | 231 | 53 |  | 503 | 293 |
| Townsend Deprivation Index, % |  |  |  |  |  |
| Low | 5910(33.1) | 1432(32.9) |  | 11044(32.9) | 6439(34.1) |
| Intermediate | 5954(33.3) | 1486(34.2) |  | 11046(32.9) | 6433(34.0) |
| High | 6013(32.6) | 1429(32.9) |  | 11476(34.2) | 6010(31.9) |
| Missing | 27 | 6 |  | 37 | 21 |
| Family history of breast cancer, % |  |  |  |  |  |
| Yes | 1665(9.3) | 395(9.1) |  | 3664(10.9) | 2125(11.2) |
| No | 16239(90.7) | 3958(90.9) |  | 29939(89.1) | 16778(88.8) |
| History of mammograms, % |  |  |  |  |  |
| Yes | 4860(27.3) | 1118(25.8) |  | 31707(94.4) | 18090(95.7) |
| No | 12958(72.7) | 3222(74.2) |  | 1873(5.6) | 806(4.3) |
| Missing | 86 | 13 |  | 23 | 7 |
| Oral contraceptive use, % |  |  |  |  |  |
| Yes | 15943(89.2) | 3872(89.1) |  | 27091(80.8) | 14741(78.1) |
| No | 1921(10.8) | 472(10.9) |  | 6438(19.2) | 4123(21.9) |
| Missing | 40 | 9 |  | 74 | 39 |
| HRT use, % |  |  |  |  |  |
| Yes | — | — |  | 14880(44.4) | 9100(48.2) |
| No | 17605(100) | 4271(100) |  | 18643(55.6) | 9766(51.8) |
| Missing | 299 | 82 |  | 80 | 37 |
| Number of births |  |  |  |  |  |
| Nulliparous | 4932(27.6) | 1217(28.0) |  | 6349(18.9) | 3245(17.2) |
| < 3 | 9788(54.7) | 2429(55.8) |  | 19595(58.3) | 11339(60.0) |
| ≥ 3 | 3170(17.7) | 704(16.2) |  | 7659(22.8) | 4319(22.8) |
| Missing | 14 | 3 |  |  |  |
| Age at menarche, years |  |  |  |  |  |
| < 12 | 2100(12.1) | 543(12.9) |  | 6181(18.4) | 3189(16.9) |
| 12-13 | 7950(45.8) | 1873(44.4) |  | 14587(43.4) | 7850(41.5) |
| ≥ 14 | 7304(42.1) | 1798(42.7) |  | 12835(38.2) | 7864(41.6) |
| Missing | 550 | 139 |  | 18 | 14 |
| Age at menopause, years |  |  |  |  |  |
| < 50 | — | — |  | 13368(39.8) | 7472(39.5) |
| 50-55 | — | — |  | 16059(47.8) | 8910(47.1) |
| ≥ 55 | — | — |  | 4168(12.4) | 2519(13.3) |
| Missing | — | — |  | 8 | 2 |

Abbreviations: BF, body fat; HRT, Hormone replacement therapy; BMI, body mass index; PBF, percent body fat

^a^ Normal weight obesity (NWO) was defined as participants with a normal BMI but an excess PBF (>33.3%). Normal weight lean (NWL) was defined as participants with a normal BMI and a normal PBF (≤ 33.3%).

^b^ *P* values < 0.05 for all variables listed in the two groups, except for Townsend deprivation index, family history of breast cancer and oral contraceptive use in premenopausal females; ethnic and family history of breast cancer in postmenopausal females.

**Supplementary Table 3** Population Attributable Risk Proportions (PARP) for postmenopausal breast cancer in the UK Biobank

| **Variable** | **PARP (95% CIs)** |
| --- | --- |
| Normal weight obesity | 6.40 (2.80 to 10.04) |
| Family history of breast cancer | 4.81 (2.98 to 6.88) |
| Hormone replacement therapy | 6.83 (2.23 to 11.34) |
| Age at menopause (≥ 50 years) | 12.41 (6.48 to 18.2) |
| Nulliparity | 2.15 (-1.67 to 6.67) |
| Oral contraceptive use | 3.83 (-6.81 to 13.1) |

The population prevalence and relative risk of each variable were estimated to calculate the population-attributable risk proportions. Models were adjusted for all covariates listed in Supplementary Table 2.

**Supplementary Table 4** The association between normal weight obesity (NWO) and postmenopausal breast without and with adjustment for NWO-related biomarkers

| Model | HR (95%CIs) | Attenuation of HR |
| --- | --- | --- |
| Base model | 1.187 (1.076-1.310) | — |
| Base model + CRP | 1.151 (1.041-1.272) | 19.25% |
| Base model + monocyte count | 1.180 (1.071-1.305) | 3.74% |
| Base model + neutrophil count | 1.166 (1.054-1.286) | 11.23% |
| Base model + β-Hydroxybutyrate | 1.170 (1.059-1.293) | 9.09% |

Based model: adjusted for age, race, smoking status, drinking status, physical activity, education level, Townsend deprivation index, family history of breast cancer, history of mammograms, oral contraceptive use, hormone replacement therapy, age at first birth, number of births, age at menarche, and age at menopause.

**Supplementary Table 5** Association of normal weight obesity (NWO) and breast cancer incidence by polygenic risk score (PRS) category after excluding participants whose follow-up times < 2 years (n=51264)

| PRS category | NWL | NWO | *P* value | *P*-interaction |
| --- | --- | --- | --- | --- |
| Overall |  |  |  |  |
| Model 1 | 1.00 | 1.187(1.065-1.324) | 0.002 | — |
| Model 2 | 1.00 | 1.185(1.062-1.322) | 0.002 | — |
| Model 3 | 1.00 | 1.185(1.062-1.321) | 0.002 | — |
| Low |  |  |  |  |
| Model 1 | 1.00 | 1.065(0.817-1.388) | 0.640 | 0.025 |
| Model 2 | 1.00 | 1.095(0.829-1.447) | 0.521 | 0.024 |
| Model 3 | 1.00 | 1.095(0.829-1.445) | 0.523 | 0.026 |
| Intermediate |  |  |  |  |
| Model 1 | 1.00 | 1.070(0.880-1.301) | 0.498 |  |
| Model 2 | 1.00 | 1.081(0.877-1.332) | 0.467 |  |
| Model 3 | 1.00 | 1.078(0.875-1.328) | 0.481 |  |
| High |  |  |  |  |
| Model 1 | 1.00 | 1.286(1.108-1.492) | 0.001 |  |
| Model 2 | 1.00 | 1.291(1.112-1.500) | 0.001 |  |
| Model 3 | 1.00 | 1.291(1.112-1.499) | 0.001 |  |

Model 1: adjusted for age, race, and the first 5 principal components of ancestry and genotyping batch.

Model 2: further adjusted for smoking status, drinking status, physical activity, education level, and Townsend deprivation index.

Model 3: further adjusted for family history of breast cancer, history of mammograms, oral contraceptive use, hormone replacement therapy, age at first birth, number of births, age at menarche, and age at menopause.

**Supplementary Table 6** Association of normal weight obesity (NWO) and breast cancer incidence by polygenic risk score (PRS) category among participants with complete covariates information (n=40824)

| PRS category | NWL | NWO | *P* value | *P*-interaction |
| --- | --- | --- | --- | --- |
| Overall |  |  |  |  |
| Model 1 | 1.00 | 1.175(1.051-1.314) | 0.005 | — |
| Model 2 | 1.00 | 1.158(1.035-1.296) | 0.011 | — |
| Model 3 | 1.00 | 1.155(1.032-1.292) | 0.012 | — |
| Low |  |  |  |  |
| Model 1 | 1.00 | 1.103(0.829-1.468) | 0.499 | 0.057 |
| Model 2 | 1.00 | 1.078(0.809-1.437) | 0.608 | 0.057 |
| Model 3 | 1.00 | 1.083(0.812-1.443) | 0.588 | 0.059 |
| Intermediate |  |  |  |  |
| Model 1 | 1.00 | 1.064(0.860-1.316) | 0.570 |  |
| Model 2 | 1.00 | 1.051(0.849-1.301) | 0.649 |  |
| Model 3 | 1.00 | 1.051(0.848-1.302) | 0.650 |  |
| High |  |  |  |  |
| Model 1 | 1.00 | 1.257(1.081-1.463) | 0.003 |  |
| Model 2 | 1.00 | 1.241(1.066-1.445) | 0.005 |  |
| Model 3 | 1.00 | 1.238(1.063-1.442) | 0.006 |  |

Model 1: adjusted for age, race, and the first 5 principal components of ancestry and genotyping batch.

Model 2: further adjusted for smoking status, drinking status, physical activity, education level, and Townsend deprivation index.

Model 3: further adjusted for family history of breast cancer, history of mammograms, oral contraceptive use, hormone replacement therapy, age at first birth, number of births, age at menarche, and age at menopause.

**Supplementary Table 7** Association between normal weight obesity (NWO) defined by percent body fat ≥ 30%, 35% and 37% and incident postmenopausal breast cancer

|  | Normal weight lean | Normal weight obesity | *P* value |
| --- | --- | --- | --- |
|  | Percent body fat ≥ 30% | | |
| Case/n | 496/18085 | 1172/34421 |  |
| Model 1 | 1.00 | 1.221 (1.098-1.357) | <0.001 |
| Model 2 | 1.00 | 1.218 (1.096-1.353) | <0.001 |
|  | Percent body fat ≥ 35% | | |
| Case/n | 1241/40686 | 427/11820 |  |
| Model 1 | 1.00 | 1.166 (1.044-1.302) | 0.007 |
| Model 2 | 1.00 | 1.163 (1.041-1.299) | 0.008 |
|  | Percent body fat ≥ 37% | | |
| Case/n | 1484/47506 | 184/5000 |  |
| Model 1 | 1.00 | 1.162 (1.005-1.356) | 0.040 |
| Model 2 | 1.00 | 1.157 (1.001-1.351) | 0.045 |

Model 1: adjusted for age, race, smoking status, drinking status, physical activity, education level, and Townsend deprivation index.

Model 2: further adjusted for family history of breast cancer, history of mammograms, oral contraceptive use, hormone replacement therapy, age at first birth, number of births, age at menarche, and age at menopause.


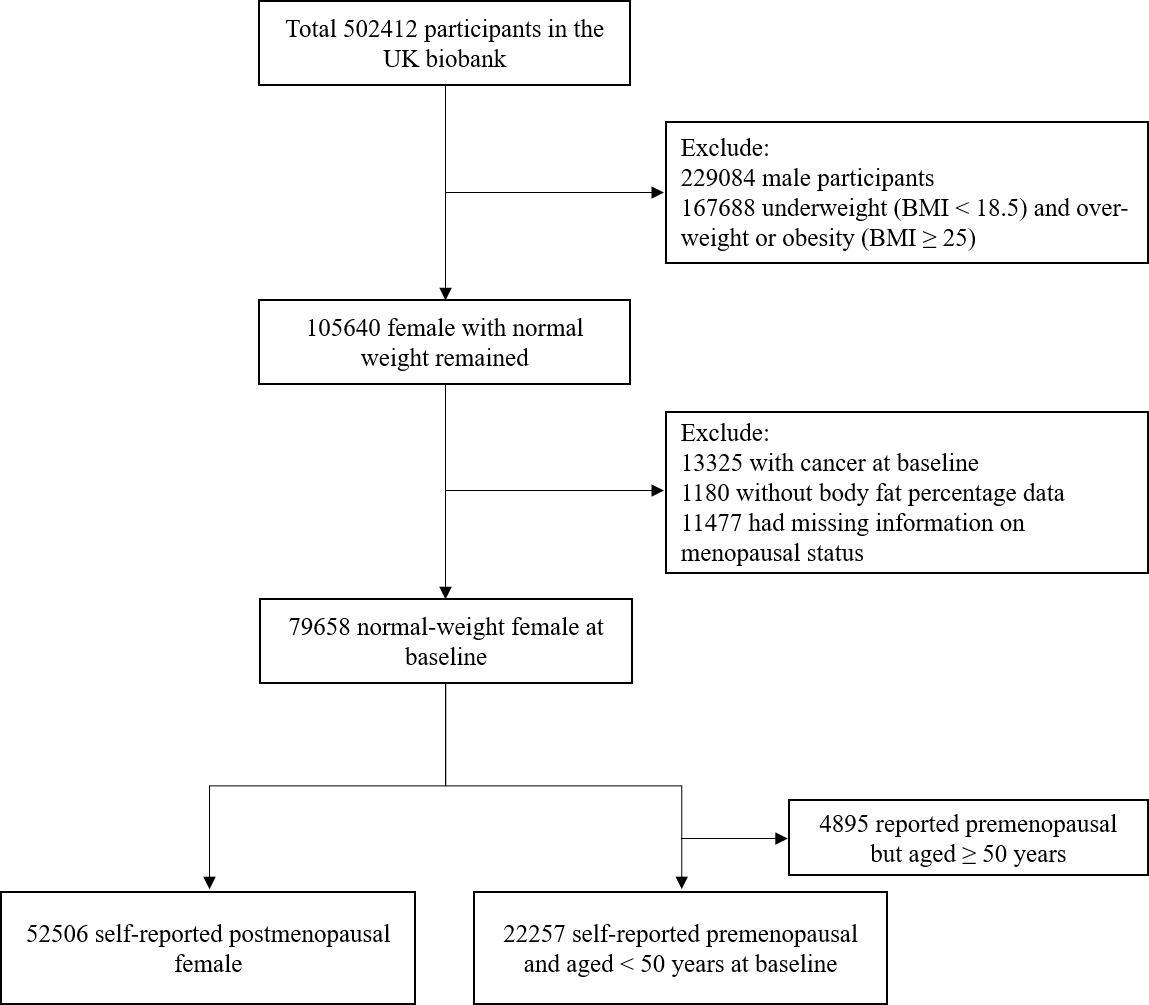


**Supplementary Figure 1** The flow chart of participants selection in the UK biobank


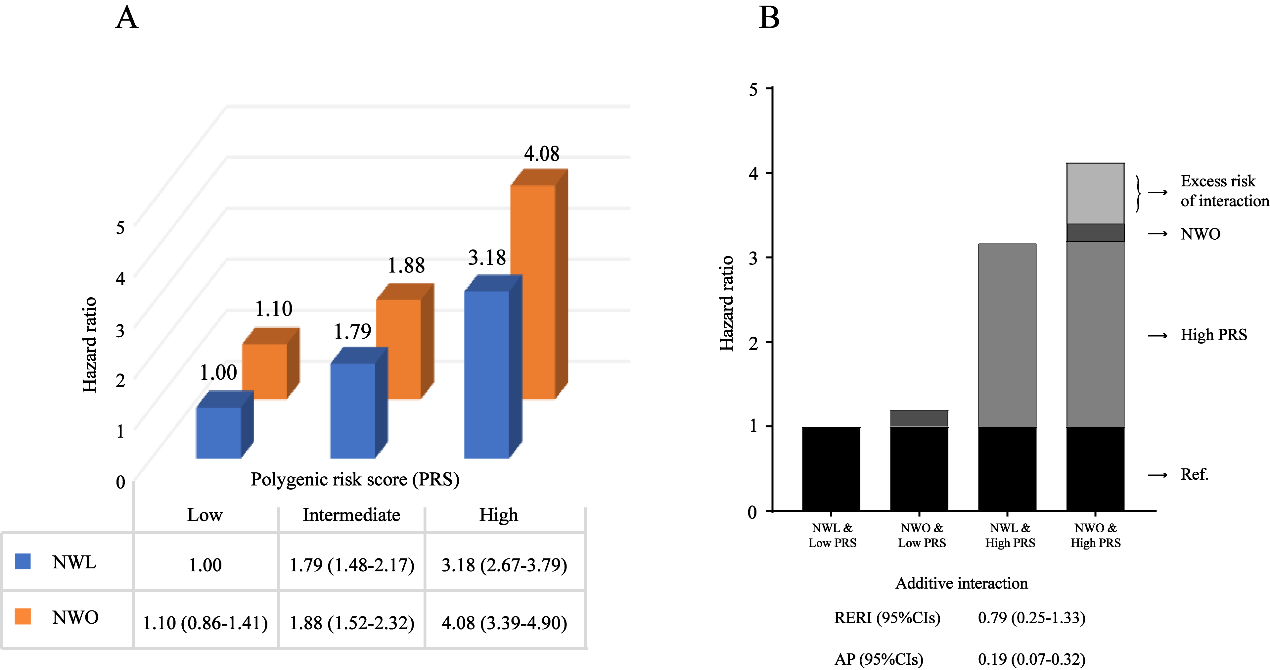


**Supplementary Figure 2** Risk of breast cancer incidence according to normal weight obesity (NWO) and Polygenic risk score (PRS) among postmenopausal women **(A)**. The model adjusted for all covariates listed in Supplementary Table 2. The additive interaction between NWO and PRS score on breast cancer incidence **(B)**. NWL, Normal weight lean; RERI, Relative excess risk due to interaction; AP, Attributable proportion due to interaction.
